# The use of intravenous versus subcutaneous monoclonal antibodies in the treatment of severe asthma: a review

**DOI:** 10.1186/s12931-018-0859-z

**Published:** 2018-08-16

**Authors:** Andrea Matucci, Alessandra Vultaggio, Romano Danesi

**Affiliations:** 1 Immunoallergology Unit, AOU Careggi, University of Florence, Largo Brambilla 3, 50134 Florence, Italy; 2 Clinical Pharmacology and Pharmacogenetics Unit, Department of Clinical and Experimental Medicine, University of Pisa, Pisa, Italy

**Keywords:** Eosinophil, Mepolizumab, Monoclonal antibodies, Omalizumab, Reslizumab, Severe asthma

## Abstract

**Background:**

Monoclonal antibodies (mAbs) approved for use as add-on therapy in patients with severe asthma target the underlying pathogenesis of asthma.

**Main body:**

Omalizumab binds immunoglobulin E (IgE), thereby inhibiting its interaction with the high-affinity IgE receptor and reducing the quantity of free IgE available to trigger the allergic cascade. Anti-interleukin (IL)-5 mAbs mepolizumab, benralizumab and reslizumab block the interaction between IL-5 and its receptor on eosinophils, thus targeting the eosinophilic pathway in asthma. Most mAbs are available as intravenous (IV) or subcutaneous (SC) formulations, as their high molecular weight and gastric degradation preclude oral administration. This review compares the pharmacology, efficacy, immunogenicity, injection- and infusion-related adverse drug reactions of subcutaneously administered omalizumab and mepolizumab with the intravenously administered reslizumab. In terms of pharmacokinetics, IV route of administration appears to be superior to the SC route due to quicker absorption, greater bioavailability, shorter time to maximum serum concentration and similar elimination half-life. Route of administration does not appear to translate into striking differences in efficacy and safety of mAbs used for the treatment of severe asthma, as all are generally considered to be effective and well tolerated. Hypersensitivity and administration-related reactions have been described with both IV and SC mAbs.

**Conclusion:**

mABs are effective and have low immunogenicity due to their nature as humanised antibodies. Evidence on the use of mAbs in indications other than severe asthma suggest that both the SC and the IV routes of administrations have their respective advantages and disadvantages; but their full utility remains to be elucidated.

## Background

Asthma is an inflammatory chronic disease of multifactorial aetiology, including allergic asthma, which is characterized by eosinophilic airway inflammation [1], often sustained by allergic sensitisation. Asthma is typically associated with bronchial hyper-responsiveness to direct and indirect triggers [1]. Severe asthma is defined as asthma that requires high-dose inhaled corticosteroids (ICS) plus a second controller (usually a long-acting β_2_ agonist [LABA]) and/or systemic corticosteroids for adequate control, or which is uncontrolled despite this high-intensity treatment [2]. An estimated 5–10% of all patients with asthma are believed to have severe refractory asthma [3].

For patients with severe uncontrolled asthma, four monoclonal antibodies (mAbs) against immunoglobulin E (IgE) or interleukin (IL)-5 are available for clinical use by either subcutaneous (SC) or intravenous (IV) administration as an add-on to ICS plus LABA therapy. The anti-IgE antibody omalizumab has been available for SC use in the USA since 2003 [4], and has also been approved in Europe since 2009 [5]. The anti-IL-5 antibodies mepolizumab and benralizumab for SC administration and reslizumab for IV administration were approved in 2015, 2017 and 2016, respectively, in both the USA [6–8] and Europe [9–11].

According to the Global Initiative for Asthma (GINA) 2017 guidelines, add-on therapy with omalizumab should be considered in patients aged ≥6 years with severe allergic asthma, while mepolizumab and reslizumab may be beneficial in patients with severe eosinophilic asthma aged ≥12 years [1]. However, it is important to note that reslizumab has only been approved for adults (≥18 years) [9].

This review provides an overview of the available IV and SC mAbs for use in patients with severe asthma and discusses the potential advantages and disadvantages associated with these routes of administration for mAbs.

### Pharmacokinetics

mAbs are made of two identical heavy and light chains held together by disulphide bonds at the hinge region to form a Y-shaped structure [12]. They were first created as mouse mAbs using hybridoma technology, but to minimise the immunogenic mouse components, murine mAbs were replaced by chimeric, humanised and then fully human mAbs [13].

The pharmacokinetics (PK) of mAbs is characterised by low distribution to the extravascular compartment, because of their large molecular size and long elimination half-life (t_1/2_), which depends on slow intracellular catabolism, with no urinary excretion [12, 14].

The most common route of administration of mAbs is IV, followed by SC administration, although intramuscular injection is also possible [12]. Oral administration is precluded because of their large molecular size and gastrointestinal degradation [14]. The rate and extent of absorption varies between mAbs and between individuals for the same mAb [12]. For mAbs administered via the SC route, absorption into the systemic circulation first requires convective transport of the mAb through the interstitial space into the lymphatic system [12]. In addition, lymphatic fluid flow is slow compared with blood, thus absorption of mAbs after SC administration is a slow process, with average time to peak concentration (T_max_) of 6–8 days [12].

In addition, PK parameters may be influenced by patient-specific factors. For example, most mAbs are administered on the basis of body weight, which is often reported to be a significant factor influencing the volume of distribution (V_d_) and/or systemic clearance (CL_s_), although this is not true for every mAb [14].

The route of administration of mAbs influences their peak serum concentration (C_max_), and this may influence the time to clinical response [15]. The first available mAb for asthma, omalizumab, was administered via the SC route [4], but in later clinical trials with anti-IL-5 mAbs, IV administration was used. Overall, T_max_ is longer with SC than IV administration (Table 1), but SC administration allows for the maintenance of more stable levels of the biological agent during the dosing interval. Following SC administration, PK profiles are approximately linear for omalizumab (at doses > 0.5 mg/kg) [4, 5] and mepolizumab (at doses 12.5–250 mg) [7, 10]. SC administration of omalizumab or mepolizumab is associated with T_max_ of up to 8 days after a single dose [4, 5, 7, 10]. In contrast, C_max_ is rapidly reached after IV administration of mepolizumab or reslizumab, generally occurring at the end of the infusion (Table 1) [6, 9, 16]. After IV reslizumab, serum concentrations typically show a biphasic decrease after C_max_ [6, 9] and PK is dose-proportional at doses of 0.3–3.0 mg/kg [9].

**Table 1:** Summary of pharmacokinetic profiles for omalizumab [4, 5], mepolizumab [7, 10, 16] and reslizumab [6, 9]

	Omalizumab	Mepolizumab		Reslizumab
Route	SC	SC	IV	IV
Absolute bioavailability	100%	100%	–	–
T_max_	7–8 days	4–8 days	End of infusion	End of infusion
V_d_	78 mL/kg	51 mL/kg	55–85 mL/kg	≈5 L
CL_S_	2.4 mL/kg/day	3.1 mL/kg/day	1.9–3.3 mL/kg/day	≈7 mL/hour
t_½_	26 days	16–22 days	≈20 days	≈24 days

*CL*_*S*_ systemic clearance, *IV* intravenous, *PK* pharmacokinetics, *SC* subcutaneous, *t*_*½*_ elimination half-life, *T*_*max*_ time to C_max_, *V*_*d*_ volume of distribution

Comparisons of the influence of route of administration on PK for mAbs approved for other disease indications also suggest broadly similar distribution and elimination with IV versus SC administration. In patients with follicular lymphoma, SC administration of the anti-CD20 antibody rituximab was shown to have non-inferior PK (meeting the prespecified C_trough_ 90% confidence interval [CI] lower limit of 0.8) to IV rituximab [17] and similar efficacy and tolerability [18]. SC trastuzumab for human epidermal growth factor receptor 2 (HER2)-positive breast cancer had non-inferior PK (meeting the prespecified margin for the difference between groups in C_trough_ of 0.8) and efficacy (meeting the prespecified margin for the difference between groups in pathological complete remission of − 12.5%), compared with IV administration, in a phase III, randomised, open-label trial in patients with early HER2-positive breast cancer [19].

SC treatment is usually given as a fixed dose, as is the case for mepolizumab [7, 10], while IV reslizumab dose varies based on the patient’s body weight [6, 9]. Indeed, in a reslizumab population PK model, heavier body weight was associated with more rapid CL_s_ and a larger V_d_, supporting the appropriateness of the recommended weight-based dosing (3 mg/kg), which produces comparable exposure across the entire range of weights [20]. Modelling of body weight-based dosing using data pooled from eight reslizumab clinical trials showed that steady-state reslizumab exposure after IV administration, including area under the drug concentration-time curve (AUC), C_max_ and average serum concentration (C_avg_), remained consistent across a wide range of patient body weights [21]. Thus, weight-based IV regimens of reslizumab represent an approach that is appropriate for individual patient’s requirements.

### Clinical efficacy

The availability of biological agents has changed the treatment strategies for severe asthma. Initially, the availability of omalizumab changed the treatment of asthma related to IgE-mediated allergic pathogenesis, and more recently, the availability of drugs targeting IL-5, which is a fundamental factor in the differentiation, activation and survival of eosinophils, changed the treatment strategy for the eosinophilic phenotype [22]. Efficacy results for these three mAbs in severe allergic asthma have been widely confirmed by various clinical trials (Table 2) and, at least for omalizumab, have been available for over ten years, including from real-world studies [23, 24]. Their clinical efficacy represents their capacity to interfere in the pathogenic mechanisms of the disease, albeit in different ways (IL-5 vs IgE).

**Table 2:** Main clinical outcomes from RCTs of omalizumab, mepolizumab and reslizumab in patients with severe asthma

First author, year (study name)	Patients	Study treatment	Main clinical outcomes
Omalizumab			
Busse, 2001 [25]	Severe allergic asthma requiring daily ICS (*n* = 525)	SC omalizumab q2w or q4w vs PBO for 28 wks	Significantly fewer asthma exacerbations per patient vs PBO with stable ICSs (0.28 vs 0.54; *p* = 0.006) and during ICS reduction (0.39 vs 0.66; *p* = 0.004)
Solèr, 2001 [26]	Symptomatic allergic asthma on daily ICS (*n* = 546)	SC omalizumab q2w or q4w vs PBO for 28 wks	Significantly fewer asthma exacerbations per patient vs PBO with stable ICSs (0.28 vs 0.66; *p* < 0.001) and during ICS reduction (0.36 vs 0.75; *p* < 0.001)
Holgate, 2014 [30]	Severe allergic asthma on ICS (*n* = 246)	SC omalizumab q2w or q4w vs PBO for 32 wks	Significantly greater median reductions in ICS dose vs PBO (60% vs 50%; *p* = 0.003); ≥50% ICS dose reduction achieved by 73.8% vs 50.8% of patients (*p* = 0.001)
Mepolizumab			
Pavord, 2014 (DREAM) [67]	Severe eosinophilic asthma (*n* = 621)	IV mepolizumab 75, 250 or 750 mg q4w vs PBO for 13 doses	Fewer clinically significant exacerbations per patient-year vs PBO (1.15–1.46 vs 2.40; *p* < 0.001)
Ortega, 2014 (MENSA) [32]	Severe eosinophilic asthma on high-dose ICS (*n* = 576)	IV mepolizumab 75 mg q4w or SC mepolizumab 100 mg q4w vs PBO for 32 wks	Significantly decreased rate of exacerbations with IV (by 47%) and SC (by 53%) mepolizumab vs PBO (*p* < 0.001); significantly greater mean increases in FEV_1_ with IV (by 100 mL) and SC (by 98 mL) vs PBO (*p* < 0.05); significantly decreased rate of exacerbations needing hospitalisation with SC mepolizumab vs PBO (by 61%; *p* = 0.02)
Bel, 2014 (SIRIUS) [31]	Severe eosinophilic asthma on systemic corticosteroids (*n* = 135)	SC mepolizumab 100 mg q4w vs PBO for 20 wks	Significantly greater likelihood of reducing systemic corticosteroid dose vs PBO (OR 2.39; 95% CI 1.25–4.56; *p* = 0.008); significantly greater median dose reduction vs PBO (50% vs 0%; *p* = 0.007); significantly lower rate of exacerbations per year vs PBO (1.44 vs 2.12; *p* = 0.04)
Chupp, 2017 (MUSCA) [33]	Severe eosinophilic asthma on high-dose ICS (*n* = 274)	SC mepolizumab 100 mg q4w vs PBO for 24 weeks	Significantly improved SGRQ total score vs PBO (LSM change from baseline–15.6 vs − 7.9; *p* < 0.0001)
Reslizumab			
Castro, 2015 [37]	Inadequately controlled asthma with ≥400/μL blood eosinophils (*n* = 953 in two duplicate studies)	IV reslizumab 3.0 mg/kg q4w vs PBO for 52 wks	Significantly reduced rate of exacerbations vs PBO (study 1 RR 0.50; 95% CI 0.37–0.67; study 2 RR 0.41; 95% CI 0.28–0.59; both p < 0.001); significantly greater increases in FEV_1_ vs PBO (study 1 RR 0.126; 95% CI 0.06–0.188; p < 0.0001; study 2 RR 0.090; 95% CI 0.003–0.153; *p* = 0.0057); significantly improved scores on the ACQ and AQLQ vs PBO (study 1 both p < 0.001; study 2 *p* < 0.001 and *p* < 0.01, respectively)
Bjermer, 2016 [38]	Inadequately controlled asthma with ≥400/μL blood eosinophils (*n* = 315)	IV reslizumab 0.3 or 3.0 mg/kg q4w vs PBO for 16 wks	Significant greater increases in FEV_1_ with 0.3 mg/kg (by 115 mL) and 3.0 mg/kg (by 160 mL) vs PBO (both *p* < 0.05); significantly improved scores on the ACQ and AQLQ vs PBO (*p* < 0.05)

*ACQ* Asthma Control Questionnaire, *AQLQ* Asthma Quality of Life Questionnaire, *CI* confidence interval, *FEV*_*1*_ forced expiratory volume over 1 s, *ICS* inhaled corticosteroids, *IV* intravenous, *LSM* least squares mean, *OR* odds ratio, *PBO* placebo, *q2w* every 2 weeks, *q4w* every 4 weeks, *RCT* randomised controlled trial, *RR* rate ratio, *SC* subcutaneous, *SGRQ* St George’s Respiratory Questionnaire

#### Omalizumab

Omalizumab is a humanised IgG1 mAb. Omalizumab selectively binds to IgE, inhibiting its interaction with the high-affinity IgE receptor (Fc epsilon R [FcεRI]) on the surface of basophils and mast cells, reducing the quantity of free IgE available to trigger the allergic cascade [4, 5]. In patients with allergic asthma, SC omalizumab for 24 weeks was associated with an 89–99% reduction in serum free IgE levels [25, 26]. Importantly, other cells such as dendritic cells (DC) express FcεRI [27], and can use this pathway to capture allergens. FcεRI/IgE-dependent allergen presentation may therefore critically lower the atopic individual’s threshold for an allergen-specific T-cell response [28]. In this connection, the therapeutic potential of anti-IgE mAbs is related to significant reductions in CD4^+^ and CD8^+^ T lymphocytes and B lymphocyte counts and central cytokine IL-4 production [29], which are involved in the pathogenesis of bronchial asthma. Furthermore, randomised controlled studies of patients with severe asthma have shown improved disease control with SC omalizumab and have also allowed for significant reductions in daily ICS requirements (Table 2) [25, 26, 30].

#### Anti-IL-5 antibodies

The therapeutic effect of mepolizumab, benralizumab and reslizumab is related to their capacity to bind with high affinity to IL-5 (dissociation constants in vitro of 100 and 81 pmol/L, respectively) and thus to block the interaction between IL-5 and its receptor on the surface of eosinophils [6, 7].

##### Mepolizumab

Mepolizumab is an IgG1 kappa mAb. In patients with asthma and blood eosinophil counts > 300 cells/μL, dose- and time-dependent decreases in blood eosinophil counts were observed with IV mepolizumab 75 mg and SC mepolizumab 125 or 250 mg administration [16]. However, authors concluded that the route of administration did not affect the drug exposure-response relationship [16]. Furthermore, given that the reduction in blood eosinophils was evident as soon as three days from administration of the drug, whether SC or IV [16], there appears to be little significant difference in the PK-pharmacodynamic profile between the two routes of administration.

The biological effects of SC mepolizumab have translated into clinical therapeutic efficacy. SC mepolizumab was associated with a significant corticosteroid-sparing effect in patients with severe eosinophilic asthma who required daily oral corticosteroids for asthma control in the SIRIUS study [31]. The MENSA clinical trial results suggested an advantage of the SC over the IV formulation. In fact, the rate of exacerbations was reduced by 47% among patients receiving IV mepolizumab and by 53% among those receiving SC mepolizumab. In addition, emergency visits or hospitalisations were reduced by 32% and 61% in the group receiving IV and SC mepolizumab, respectively. The improvement in the Asthma Control Questionnaire-5 (ACQ-5) score was 0.42 points and 0.44 points greater in the two mepolizumab groups, respectively, than in the placebo group [32]. SC mepolizumab was also associated with an improvement in health-related quality of life, with the results of the phase IIIb MUSCA demonstrating significant improvements in St George’s Respiratory Questionnaire scores with SC mepolizumab versus placebo after 24 weeks of treatment [33].

##### Benralizumab

Benralizumab is a humanised, afucosylated, monoclonal antibody (IgG1, kappa) that directly binds to the alpha subunit of the human interleukin-5 receptor (IL-5Rα). The IL-5 receptor is expressed on the surface of eosinophils and basophils. In an in vitro setting, the absence of fucose in the Fc domain of benralizumab facilitates binding to FC gamma R (FcɣRIII) receptors on immune effectors cells, such as natural killer (NK) cells, leading to apoptosis of eosinophils and basophils through antibody-dependent cell-mediated cytotoxicity (ADCC). Benralizumab is indicated as an add-on maintenance treatment in adult patients with severe eosinophilic asthma inadequately controlled despite high-dose ICS plus LABA. Benralizumab is administered at the dose of 30 mg by SC injection given every 4 weeks for the first 3 doses, then every 8 weeks. In patients with an eosinophil count of ≥300 cells/μL, significant improvements in lung function, as defined by forced expiratory volume over 1 s (FEV_1_), were observed. At the end of treatment, benralizumab patients had a 24% improvement from mean baseline in FEV_1_ of 1.66 L. In addition, studies also showed greater improvements in FEV_1_ in patients with higher baseline blood eosinophil counts and more frequent prior exacerbation history [34]. Interestingly, patients receiving benralizumab achieved greater reductions in daily maintenance oral corticosteroid dose, while maintaining asthma control [35].

##### Reslizumab

Reslizumab is an IgG4 kappa mAb. The biological activity of reslizumab has been described in patients with severe persistent asthma treated with high-dose ICS or oral corticosteroids, in which a single dose of IV reslizumab 0.3 or 1 mg/kg reduced peripheral eosinophil counts from baseline in a dose-dependent manner after 48 h [36]. Compared with placebo, the decrease in blood eosinophil counts with reslizumab 1 mg/kg remained significant up to day 30 after administration (*p* = 0.05 vs placebo) [36].

These biological effects correspond with clinical results for asthma control with IV reslizumab. In patients with inadequately controlled asthma and elevated blood eosinophil levels (≥400 cells/μL), IV reslizumab 3.0 mg/kg every 4 weeks was associated with a significant reduction in the frequency of asthma exacerbations, as well as significant improvements in FEV_1_, asthma quality of life (AQLQ) and ACQ-7 scores, in two identically designed phase III studies [37]. In another phase III study, which evaluated reslizumab 0.3 and 3 mg/kg every 4 weeks, both doses were associated with significant improvements in FEV_1_ and ACQ scores, as well as reductions short acting β-agonist use [38]. Reslizumab has consistently reduced blood eosinophil concentrations in phase III studies [37, 38].

Several post hoc analyses have been conducted to identify the subgroups of traditionally difficult-to-treat patients who may respond to reslizumab treatment. These groups include patients with chronic sinusitis and nasal polyps, asthma severity corresponding to step 4 and 5 of the GINA classification, oral corticosteroid-dependent patients and those aged ≥65 years [37, 39, 40]. In addition, post hoc analyses of phase III clinical trials indicate that patients who experience FEV_1_ response (≥100 ml) and/or ACQ response within the first 16 weeks have greater improvements in the clinical asthma exacerbation rate that those who do not [41]. Furthermore, an algorithm that takes into account the FEV_1_, ACQ, asthma quality of life and clinical asthma exacerbation rate at baseline and at week 16 has been developed to predict early response to reslizumab and help guide treatment decisions [42].

Overall, the main clinical outcomes from the key clinical studies of omalizumab, mepolizumab and reslizumab are summarised in Table 2. These data indicate that mAbs are effective in the treatment of severe asthma. While most outcomes appear to be similarly improved by drugs with SC and IV route of administration, it should be noted that IV reslizumab has been associated with improvement in FEV_1_, ACQ-7 and Asthma Symptom Utility Index (ASUI) at first assessment, which occurred 4 weeks after treatment initiation [37]. On the other hand, no mAb administered subcutaneously has been shown to have such rapid effects. Relatively slow absorption and lower bioavailability of SC mAbs fits well with the current knowledge of the mechanisms underlying their distribution, namely reliance on the lymphatic system.

### Immunogenicity

Biological agents have the potential to induce an immune response leading to the production of specific anti-drug antibodies (ADAs). ADAs are particularly relevant when they neutralise the drug [43, 44], because they bind directly to the active site of the mAb, eliminating the interaction between the drug and its target and, therefore, reducing the clinical therapeutic effect [45]. Potential clinical consequences of immunogenicity range from little or no effect (as discussed below) to altered PK or efficacy, as well as induction of hypersensitivity reactions [45]. Infusion reactions have also been linked to the formation of drug-ADA complexes [46].

#### Immunogenicity in severe asthma

In comparison with the immunogenicity of the mAbs for rheumatoid arthritis adalimumab and infliximab (mentioned earlier), mAbs used for severe asthma appear to present less of a problem. Humanised mAbs such as omalizumab, mepolizumab and reslizumab, are mostly derived from human Ig sequences, with only the complementarity-determining regions being of murine Ig origin [12]. There were no cases of anti-omalizumab antibody development with SC omalizumab administration in patients with severe allergic asthma [25, 26, 30, 47]. During mepolizumab treatment in the MENSA trial, anti-mepolizumab antibodies were detected in 4% of patients with IV administration, 5% with SC administration and 2% with placebo; neutralising antibodies were not detected [32]. In SIRIUS, anti-mepolizumab antibodies were detected in six patients overall (4%), with one patient developing neutralising antibodies after the first dose of SC mepolizumab; however, serious adverse events relating to immunogenicity were not reported [31]. A much greater difference in immunogenicity between the SC and IV formulations of mepolizumab has been observed; in a study using a modified ADA assay where a mepolizumab competitive anti-IL-5 antibody was added in a pre-treatment step, the ADA rate for IV and SC mepolizumab was 1% and 8%, respectively [48]. This supports previous data cited in this review suggesting greater immunogenicity of SC formulations; however, as mentioned, many factors influence the development of immunogenicity, and assay method may also result in variation in observations of immunogenicity [46].

In an analysis of pooled data from all four phase III placebo-controlled reslizumab studies in patients with asthma, 5% (53/938) of reslizumab 3 mg/kg recipients had anti-reslizumab antibodies, but readings were of low titre and frequently transient [9]. The same ADA rate was found in an open-label extension study of patients with asthma receiving IV reslizumab 3 mg/kg for up to 36 months (5% [49/1014]) [9]. Among patients positive for treatment-emergent anti-reslizumab antibodies, the reduction in blood eosinophil levels was similar to that observed in patients negative for anti-reslizumab antibodies, suggesting that these antibodies were not neutralising [38]. Anti-reslizumab antibodies were not associated with hypersensitivity reactions and had no effect of the clinical pharmacodynamics or PK of reslizumab [9].

While data suggest the possibility of an immunogenic response to anti-IL-5 mAbs, the potential clinical implications in patients with asthma are not yet firmly established.

#### Factors influencing immunogenicity

Many factors influence the development of immunogenicity: drug dose, administration route, but also concomitant medications, underlying disease and the drug structure [46]. Immunogenicity of mAbs depends, at least in part, on the fraction of nonhuman sequence in the drug molecule [12]. Immunogenicity is lower with more humanised mAbs compared with chimeric mAbs [46]. Infliximab, a chimeric mAb that is administered intravenously, is associated with the formation of ADA in up to 50% of patients with rheumatoid arthritis, while even the humanised mAb adalimumab, which is administered subcutaneously, induces ADA in a significant proportions of these patients (12–37%) [46]. Nevertheless, mAbs are generally well tolerated in humans, despite containing sequences that may be recognised by the recipient as non-self epitopes and can stimulate an immune response [13].

In fact, immunogenicity appears to be associated not only with route of administration, but also with drug dose [49] and duration of therapy [12]. For example, higher doses of the tumour necrosis factor (TNF)-α blocker infliximab – corresponding to higher serum drug levels – appeared to be linked to lower immunogenicity and greater duration of effect [50]. In a study of 101 patients with active rheumatoid arthritis, ADAs formed in 53%, 21% and 7% of patients receiving infliximab 1, 3 and 10 mg/kg, respectively [50]. The reasons for this inverse relationship between mAb dose and immune response are yet to be elucidated [12]. Longer treatment duration may be associated with an increased opportunity for an immune response [12].

#### Immunogenicity in other indications

Studies in mice have indicated that the strength of an immune response towards the biological agent may be dependent on the route of administration [51, 52]. Generally, SC administration of anti-TNF-α mAbs is more immunogenic than IV administration, due to the smaller volumes used, slower drug distribution and greater variability in inter-individual drug exposure, leading to increased formation of ADAs. Another reason for greater immunogenicity of the SC route could be the fact that skin is highly specialised for processing foreign antigens [15]. Other mAb classes for which data exist comparing the immunogenicity of IV versus SC formulations include tocilizumab (IL-6 blocker) [53], trastuzumab (HER2 antagonist) [54], and rituximab (B-cell inhibitor). A meta-analysis of data from 8974 patients in 14 studies (five SC tocilizumab and nine IV tocilizumab) reported a low and similar risk of immunogenicity with the SC and IV formulations, with the proportion of patients with ADAs being 1.5% versus 1.2%, respectively [53]. In contrast, more SC recipients than IV recipients who developed ADAs were also positive for the neutralising assay (85.1% vs 78.3%), although presence of neutralising ADAs did not appear to result in loss of therapeutic efficacy of either formulation [53]. ADA formation was greater with SC trastuzumab than with IV trastuzumab in a phase III trial in 596 patients with early breast cancer comparing these formulations (14.6% vs 7.1%), although authors concluded there was no apparent significant relationship between ADA formation and efficacy or safety [54]. Finally, in the SABRINA phase III trial comparing SC vs. IV rituximab in 410 patients with follicular lymphoma, the rate of ADA formation was low and similar between groups (2% vs 1%) [18]. Importantly, the SC formulation of rituximab used in the SABRINA trial is a novel formulation designed to enable delivery of therapeutic doses at acceptable volumes of administration by using a 12 times more concentrated dose and including a recombinant human hyaluronidase in the formulation to enhance drug dispersion and absorption by temporarily hydrolysing hyaluronic acid fibres in the interstitial matrix [18], thereby overcoming the PK limitations of SC administration.

#### Differences in immunogenicity between SC and IV administration

Compared with IV administration, SC injection of large molecules such as mAbs is thought to increase the risk of immunogenicity via DC processing [45]. DCs are present in greater numbers at the cutaneous and subcutaneous layers of the skin, and are therefore located in an ideal position to capture the drug, process it and then migrate to lymph nodes, where they can act as antigen-presenting cells for autologous T cells, giving rise to the immune response [55]. In addition to migratory DCs, SC drugs may be subjected to a second round of antigen presentation by lymph node-resident DCs [56], thus producing a generally stronger immune response than IV drugs.

### Infusion reactions

The adverse effects characteristic of mAbs relate to the body’s immune response to the drug. This response may range from being a minor, transient, subclinical reaction to a serious, potentially fatal, reaction when the immune system overreacts (hypersensitivity) [57]. Infusion-related reactions to mAbs are typically mediated via ADA immune complexes [46], and may be local or systemic, the latter being more frequent, though less severe and typical of drugs administered via SC route. In patients treated with infliximab, ADAs were mostly represented by members of the IgG class [58]; however, multiple classes (IgM, IgE, IgG) and sub-classes (IgG1–4) can be produced during humoral anti-drug response [59]. In patients who experienced severe reactions to infliximab, the presence of specific circulating IgE antibodies and positive skin test to infliximab have been reported [59, 60]. In general, SC administration may be associated with localised injection-site reactions, including hypersensitivity, tenderness, warmth and redness [61], whereas both SC and IV administration may cause systemic reactions [57].

Adverse reactions highlighted in the prescribing information for the anti-IL5 mAbs mepolizumab and reslizumab and the anti-IgE mAb omalizumab include such hypersensitivity reactions and administration-related reactions [5, 9, 10], although differences in their tolerability profiles may be observed based on their immunogenicity and route of administration. For example, in patients with severe allergic asthma, local injection-site symptoms were more common with SC omalizumab (*n* = 274) than with placebo (*n* = 272) in one study (11.8% vs 7.7%); omalizumab was more frequently associated with redness, warmth and itching symptoms [26]. Most of these adverse events were of mild severity. Similar results were reported in another placebo-controlled trial of SC omalizumab in 246 patients with severe allergic asthma: most injection-site reactions were mild and transient and were reported in 20.4% of omalizumab recipients versus 10.3% of placebo recipients [30]. Severe injection-site reactions occurred with a similar incidence in both groups [30]. In a third study, authors concluded that the tolerability profile of SC omalizumab was broadly similar to that of placebo [25]. It seems overall that the problem of injection-site reactions appears to be limited for SC omalizumab.

SC mepolizumab appears to be similarly well tolerated. In the SIRIUS study in 135 patients with severe eosinophilic asthma, local injection-site reactions occurred in only four patients (6%) on SC mepolizumab 100 mg and two (3%) on placebo [31]. Four mepolizumab and three placebo recipients experienced systemic reactions (severity unspecified) [31]. One patient experienced serious type IV delayed hypersensitivity to mepolizumab, which required hospitalisation [62].

The greater frequency of injection-site reactions with the SC route versus the IV route was confirmed in the only head-to-head comparison to date of IV versus SC administration of monoclonal antibodies in patients with severe asthma (the MENSA trial) [32]. In this randomised, double-blind, double-dummy study in 576 patients with severe eosinophilic asthma, injection-site reactions were reported more often with SC mepolizumab 100 mg (9% of patients) than with IV mepolizumab 75 mg or placebo (3% for both) [32]. IV reslizumab appears to be better tolerated than IV mepolizumab with regard to injection-site reactions, with the same proportion of patients receiving reslizumab 3.0 mg/kg and placebo (2% of patients) experiencing administration site reactions in a pooled analysis of four phase III studies. None of the administration site reactions/events were severe, serious or resulted in discontinuation [9].

Although serious infusion reactions are a rare event for biological agents used in asthma, in three studies of patients with inadequately controlled asthma and elevated eosinophil levels, IV reslizumab was associated with anaphylactic reactions in three ADA-negative patients, who responded to standard treatment and were withdrawn from the studies [37, 63].

Treatment with biological agents, specifically mAbs, is associated with the formation of circulating immunocomplexes, leading to the activation of the complement system. Activation of C1 fraction occurs at significant levels only when it binds to the CH2 domains of IgG molecules. Furthermore, only certain subclasses of IgG are effective complement activators and exert this activity in human IgG1, but not IgG4. Regarding mAbs used in asthmatic patients, it is important to underline that reslizumab is an IgG4 and that it does not activate the complement system.

Besides drug efficacy and tolerability, the rational choice of mAb and route of administration is also influenced by patient preference, pharmacoeconomic considerations and other factors, as discussed below.

### Patient preference, pharmacoeconomics and other considerations

In general, selecting the appropriate route of administration should be driven by efficacy and safety, as well as cost effectiveness and patient preference, as this will ensure optimal treatment adherence [64].

As discussed previously, mAbs are often administered by the IV route, which provides maximum bioavailability with minimal risk of immunogenicity. In addition, IV administration is suitable for medications that are administered in large volumes and those that may cause irritation [65]. The drawbacks of IV administration include the fact that it often requires dedicated personnel, which likely makes it less convenient and, if performed at a hospital, more expensive [45].

SC administration is less invasive than IV administration, it may be performed by the patient and therefore, is associated with shorter clinic visits and more optimised use of healthcare resources [45, 65]. On the other hand, disadvantages of SC administration include slower absorption and lower bioavailability than IV, as well as higher probability of injection-site reactions and pain associated with administration of large fluid volumes [65].

As highlighted in a recent systematic review (encompassing any drug class and indication), there are limited data on these various factors influencing choice of injection route [64]. Thus, data for mAbs that are well-established therapeutic options in inflammatory diseases such as rheumatoid arthritis and oncology are included here.

In an observational study of 201 patients with rheumatoid arthritis who were receiving treatment with IV infusions when asked if they would switch to SC administration, patients (54.2%) who chose to switch from IV to SC administration found SC injections more convenient than continuing IV therapy [66].

Studies examining patient preference and/or adherence to IV or SC formulations of mAbs for severe asthma are required to further aid clinicians in determining the optimal drug and route of administration for their patients.

## Discussion

Of the three mAbs that are available as add-on therapy in patients with severe asthma, IV and SC routes of administration may be utilised: reslizumab for the former and mepolizumab, benralizumab and omalizumab for the latter. Route of administration influences their pharmacology, but this does not appear to translate into striking differences in efficacy and safety, with all three drugs considered efficacious and generally well tolerated.

One of the main effects of route of administration on drug PK profile is on absorption, which is slower after SC dosing compared with IV dosing [12]. Higher clinical variability between subjects was observed following SC administration compared with the IV route, which might reflect this difference in the absorption process [15]. As may be expected, the T_max_ of IV reslizumab is much shorter than SC mepolizumab, benralizumab and omalizumab. Generally, mAbs have low V_d_ and t_1/2_ because of their large molecular size and hydrophilic nature [14], with some data suggesting a longer t_1/2_ with IV versus SC administration of mepolizumab [16]. Therefore, form the PK standpoint, IV route of administration appears to be superior to the SC route due to complete bioavailability, shorter T_max_ and similar t_1/2_.

In clinical studies, reslizumab, benralizumab, mepolizumab and omalizumab have been consistently found effective in the treatment of patients with severe allergic asthma, reducing the number of clinical exacerbations experienced by patients in a year. Of the different drugs, mepolizumab has been studied for both IV and SC administration. The results of the MENSA clinical trial indicate that the SC mepolizumab may have superior efficacy over the IV formulation with regard to reducing the rate of serious asthma exacerbations [32]. On the other hand, IV reslizumab has been associated with significant and consistent improvements in lung function and patient-reported outcomes within 4 weeks as well as a reduction of serious asthma exacerbations [37].

Immunogenicity is an important component of the pharmacology of mAbs. In this respect, the IV route of administration is likely to be superior, as it is associated with lower immunogenicity, as well as fewer local hypersensitivity reactions [46]. However, all the mAbs for severe asthma have a relatively low immunogenic potential because they are humanized mAbs, and data from clinical studies of SC omalizumab, benralizumab, mepolizumab and IV reslizumab suggest little or no ADA production. In the case of IV reslizumab, the ADAs were of low titre, frequently transient, with no evidence of neutralising ADAs, and were not associated with hypersensitivity reactions [9, 38]. Pivotal clinical studies indicate that there appears to be minimal influence of ADA formation on therapeutic effect. SC omalizumab and mepolizumab and IV reslizumab are all effective in reducing asthma exacerbations in patients with the appropriate severe asthma phenotype. While hypersensitivity and administration-related reactions are characteristic of both IV and SC mAbs, SC omalizumab and mepolizumab and IV reslizumab were all generally well tolerated. In the only head-to-head comparison of IV versus SC administration of a mAb for asthma, the safety profiles of IV and SC mepolizumab were similar [32].

## Conclusions

Currently available mAbs for the treatment of severe asthma include omalizumab, mepolizumab and reslizumab. These drugs are effective and have low immunogenicity due to their nature as humanised antibodies. Data collected on mAbs used in indications other than severe asthma suggest that both the SC and the IV routes of administrations have their respective advantages and disadvantages. Further research into this area is necessary.

## Abbreviations

ADA: Anti-drug antibodies
AQLQ: Asthma quality of life
ASUI: Asthma Symptom Utility Index
AUC: Area under the concentration-time curve
CI: Confidence interval
CL_s_: Systemic clearance
C_max_: Peak serum concentration
DC: Dendritic cells
FcɣRIII: FC gamma R
FcεRI: Fc epsilon R
FEV_1_: Forced expiratory volume over 1 s
GINA: Global Initiative for Asthma
HER2: Human epidermal growth factor receptor 2
ICS: Inhaled corticosteroid
Ig: Immunoglobulin
IL: Interleukin
iv: Intravenous
LABA: Long-acting β_2_ agonist
mAbs: Monoclonal antibodies
OR: Odds ratio
PBO: Placebo
PK: Pharmacokinetics
q2w: Every 2 weeks
q4w: Every 4 weeks
RCT: Randomised controlled trial
RR: Rate ratio
SC: Subcutaneous
t_1/2_: Half-life
T_max_: Time to maximum plasma drug concentration
TNF: Tumour necrosis factor
V_d_: Volume of distribution
